# The territorialization of Severe Acute Respiratory Syndrome and its socioeconomic, demographic and public health policy risk factors in Belém, state of Pará, Eastern Amazon, Brazil: a cross-sectional and ecological study

**DOI:** 10.1371/journal.pone.0318607

**Published:** 2025-03-12

**Authors:** Nelson Veiga Gonçalves, Alessandra Lima Leal, Heloisa Maria Melo e Silva Guimarães, Arthur Carneiro Bernardes, Silvana Rossy de Brito, Taiana Moita Koury Alves, Tainara Carvalho Garcia Miranda Filgueiras, Thayse Moraes de Moraes, Matheus Pereira do Couto Rocha, Renan Faria Cardoso, Bruno Yudi Shimomaebara Sousa, Claudia do Socorro Carvalho Miranda

**Affiliations:** 1 Laboratory of Epidemiology and Geoprocessing of Amazon, University of the State of Pará (UEPA), Belém, Brazil.; 2 Programa de Pós-Graduação em Saúde Coletiva na Amazônia, Federal University of Pará (UFPA), Belém, Brazil.; 3 Programa de Pós-Graduação em Biologia Parasitária na Amazônia, University of the State of Pará (UEPA), Belém, Brazil.; 4 Cyberspace Institute, Federal Rural University of Amazon (UFRA), Belém, Brazil; 5 Programa de Pós-Graduação em Inteligência Territorial e Sustentabilidade, Centro Universitário do Pará (CESUPA), Belém, Brazil; Universidade Federal do Para, BRAZIL

## Abstract

Severe Acute Respiratory Syndrome is an important public health problem in Brazil due to the large number of cases. It has a high mortality rate related to risk factors that include systemic arterial hypertension, type 2 diabetes mellitus, male gender and advanced age. This cross-sectional and ecological study analyzed the spatial distribution of this disease related to the evolution of COVID-19 cases and their epidemiological, demographic, socioeconomic and public health policy conditions in the administrative districts of Belém, state of Pará, in the eastern Brazilian Amazon, from 2021 to 2023. Data from the Ministry of Health, the National Institute for Space Research and the Brazilian Institute of Geography and Statistics were used. The statistical and spatial analysis of the data used the chi-square test of equal expected proportions with a significance level of 0.05% and the techniques of ordinary multivariate linear regression and percentiles, with the results expressed by means of choropleth maps, using the Bioestat 5.4 and Arcgis 10.5.1 software. The epidemiological profile analyzed 3,511 cases, following the national pattern with statistical significance. The pathology was not distributed homogeneously in spatial terms and was associated with a territorial and socioeconomic segregation of the population in the neighborhoods and their administrative districts, with great differences in their demographic characteristics, living conditions and public services for treating the disease, especially when we consider the relationship between the outskirts and the center of the municipality. This has revealed unequal development, which has produced health inequalities in the study area. With that in mind, we emphasize the urgency of expanding these services in the places identified as most vulnerable, with a view to equal care access for the disease.

## Introduction

Coronavirus disease 2019 (COVID-19) is an infectious disease, the etiological agent of which is the SARS-CoV-2 virus of the *Coronaviridae* family*,* which was initially identified as causing respiratory diseases in humans and animals in the 1960s [1,2]. This pathology has an expressive infectivity, which mainly occurs by oral transmission through direct contact with droplets expelled by infected patients or indirect contact with contaminated objects and surfaces, and a pandemic was declared by the World Health Organization (WHO) in March 2020 [3,4]. Severe Acute Respiratory Syndrome (SARS) is a major public health problem because it is related to the worsening of COVID-19 and because it has a high mortality rate associated with risk factors such as Systemic Arterial Hypertension (SAH), Type 2 Diabetes Mellitus (T2DM), obesity, immunosuppression, male gender, advanced age and others [5,6].

Between 2021 and 2023, approximately 693,616,645 people were affected by COVID-19 worldwide, with 5,117,574 deaths resulting from its progression to the severe form of the disease, with the United States and China having the highest number of cases [7,8]. During this period, around 38,210,000 cases and 708,638 deaths were reported in Brazil, mainly in the Southeast and Northeast regions. The state of Pará, the most populous territory in the northern region of Brazil with 8,272,724 inhabitants, recorded around 886,000 notifications and 19,166 deaths due to complications from COVID-19 [9,10]. The municipality of Belém, the state capital, with a population of 1,492,745 inhabitants, recorded 159,882 infected people, with 5,481 deaths. The highest number of deaths from SARS occurred during the “first wave” of the disease, from February to June 2020 [10].

The municipality of Belém do Pará, located in the eastern Brazilian Amazon, has 71 neighborhoods and 39 islands distributed in 8 Administrative Districts (ADs). It covers a total area of 1,059.458 Km², with the urban area covering 250.20 Km² and the rural area 258.11 Km² [11]. The populations of these ADs have different demographic, socioeconomic and epidemiological characteristics, as well as public health policies related to assisting cases of COVID-19 with progression to its severe form, whose occurrence in different ways may be related to the combination of these factors in the districts of the municipality [12,13]. In this context, territorialized and systematic studies of the epidemiological scenario of SARS are a major challenge for epidemiology, since they enable monitoring of this disease and planning health actions aimed at mitigating it, in accordance to the Brazilian Unified Health System (SUS) especially in areas where the relationship between the above variables may suggest significant health inequities [14].

Spatial data analyses in health have thus been widely used in studies on the occurrence of diseases of various causes, with applications in monitoring and controlling their nosological scenarios [15]. This method of producing epidemiological knowledge has made it possible to characterize the distribution of health problems in the various Brazilian territories, seeking to identify vulnerabilities associated with demographic risk factors, considering the population contrasts between urban, peri-urban and rural areas, as well as socio-economic factors such as income distribution and living conditions, and public policies related to the absence of care and control institutions for the severe form of COVID-19, especially in the elderly people [16–18].

In view of the above, this study sought to contribute to the development of a systematic memory of the recent pandemic process by analyzing the spatial distribution of COVID-19 cases that evolved into SARS, associating them with epidemiological, demographic, socioeconomic and public health policy contrasts and vulnerabilities, in the ADs of the municipality of Belém, state of Pará, Eastern Amazon, from January 2021 to December 2023.

## Materials and methods

In this cross-sectional and ecological study, a population of 78,130 individuals affected by COVID-19 and 3,511 who evolved to SARS was studied, from 2021 to 2023, in the municipality of Belém, state of Pará. The spatial units of analysis used were the eight ADs of Belém: Belém Administrative District (DABEL), Guamá Administrative District (DAGUA), Icoaraci Administrative District (DAICO), Sacramenta Administrative District (DASAC), Outeiro Administrative District (DAOUT), Bengui Administrative District (DABEN), Mosqueiro Administrative District (DAMOS) and Entroncamento Administrative District (DAENT).

Epidemiological data on COVID-19 cases occurring in ADs was obtained from the Belém Municipal Health Department. The data related to SARS (location, gender, age group, ethnicity, schooling, area of residence, comorbidities, vaccine, type of hospitalization facility and evolution), considering the location of the Notifying Health Institution (NHI), was collected from the Surveillance Secretariat of the Ministry of Health (MS). Public policy data relating to all the health institutions in the municipality and those that reported the disease were obtained from the Ministry of Health National Register of Health Establishments. Territorial boundaries (neighborhoods and ADs) and demographic (population quantitative) and socio-economic (Average Municipal Human Development Index of the neighborhoods that make up the ADs - AMHDI) data were obtained from the Brazilian Institute of Geography and Statistics (IBGE). The dataset used in this work, including cartographic data, is in the public domain with unrestricted access. All of them were obtained from official Brazilian government sources.

Once the data had been collected they were debugged excluding the individuals who did not live in the municipality of Belém do Pará, as well as those with inconsistencies, incompleteness and duplicates. The data was then georeferenced in the laboratory using TerraView 5.6.3 opensource software, in order to build a Geographic Database (BDGEO). Descriptive and inferential techniques were used in the statistical analysis of the data related to the individuals’ variables, using proportions and the non-parametric chi-square test of equal expected proportions, with a significance level of p <  0.05, using the BioEstat 5.0 program (Ministry of Health, Brasília-DF, BR). With a view to performing a quantitative and territorialized analysis of COVID-19 evolving towards an unfavorable outcome of death, two population groups with specific characteristics were used: elderly persons with and without SARS.

The analysis of the spatial distribution of COVID-19 and SARS was carried out using ADs, for which thematic and choropleth vector maps were drawn up in the laboratory by the authors of this article, including six intervals related to the prevalence or otherwise of these diseases (number of cases/population x 100,000). The population quantitative of the ADs and the distribution of their AMHDI (arithmetic mean of the MHDI of their neighborhoods) were added to these maps, using the five-interval quantile division technique. The use of the stratification process for all of the above variables aimed to identify their very low, low, moderate, high and very high occurrence in the study area and period, considering different colors in accordance with the national cartographic standard, using ArcGIS 10.5.1 software (ESRI, Redlands-CA, USA).

To analyze the relationship of spatial dependence between socioeconomic, public health policy and demographic variables, as predictors of the occurrence of SARS that did not show autocorrelation in the study area and period after exploratory bivariate analysis, the ordinary least squares multivariate linear regression technique was used, which considered an adjusted *R*^*2*^ >  0.5 and an *F* with a p-value <  0.05 to be a statistically significant relationship. The GeoDa 1.22 open-access computer environment (University of Illinois, Urbana-Champaign-IL, USA) [19] was used for this.

In this study, ethical aspects were ensured in accordance with the Declaration of Helsinki, the Nuremberg Code and the norms of Resolution 466/12 of the National Health Council. As this was a study based on secondary data and unrestricted public access was provided, submission to the Research Ethics Committee was waived, according to the opinion of the Tropical Medicine Center of the Federal University of Pará, under CAAE number 61452522.9.0000.5172 and favorable opinion number 5.779.621.

## Results

This study analyzed 78,130 COVID-19 notifications made by the municipality of Belém, of which 3,511 evolved into SARS in most of its ADs, with a concentration in some neighborhoods. The prevalence of the disease was 251.97 cases per 100,000 inhabitants in the study period from 2021 to 2023, which is considered a high indicator in the Brazilian context. In analyzing the occurrence of the disease in relation to characteristics among individuals, a higher percentage was observed in males (53.23%), in the elderly (56.11%), persons with brown skin (64.77%), with unknown educational level (71.77%), living in the urban area of Belém (89.03%), with comorbidities (56.96%), unknown vaccination (62.15%), hospitalization in a public institution (62.69%) and with evolution to death (49.87%). It is worth noting that the high percentage of unknown information on educational level and vaccination is related to deficiencies in the process of properly filling in the notification form. These epidemiological profile results were statistically significant and in line with the Brazilian pattern, as can be seen in Table 1.

**Table 1:** Epidemiological profile of SARS cases, in the Administrative Districts of Belém, from 2021 to 2023.

Variable	Category	N = 3511	%	P-value^a^
**Gender**	Female	1642	46.77	0.0001
	Male	1869	53.23	
**Age Group**	Child (0-12 years old)	61	1.74	<0.0001
	Adolescent (13-17 years old)	5	0.14	
	Adult (18-59 years old)	1475	42.01	
	Elderly (>=60 years old)	1970	56.11	
**Ethnicity**	White	589	16.78	<0.0001
	Brown-skin	2274	64.77	
	Black	111	3.16	
	Ignored	537	15.29	
**Schooling**	Primary School	347	9.88	<0.0001
	High School	356	10.14	
	Superior	288	8.20	
	Ignored	2520	71.77	
**Area of Residence**	Rural	27	0.77	<0.0001
	Urban	3126	89.03	
	Ignored	358	10.20	
**Comorbidities**	No	1511	43.04	<0.0001
	Yes	2000	56.96	
**Vaccine**	No	790	22.50	<0.0001
	Yes	539	15.35	
	Ignored	2182	62.15	
**Type of Hospitalization Facility**	Private	1310	37.31	<0.0001
	Public	2201	62.69	
**Evolution**	Cure	1562	44.49	<0.0001
	Death	1751	49.87	
	Death by Other Causes	20	0.57	
	Ignored	178	5.07	

^a^*p* values were calculated using the Chi-Square test of equal expected proportions.

**Source: EPIGEO/CCBS/UEPA**

From a quantitative point of view, it was observed that the two populations studied, elderly people with COVID-19 with or without SARS, showed different patterns of distribution of this severe outcome, by AD. Thus, it was observed that the elderly population with COVID-19 cases without SARS showed the highest occurrences of progression to death and number of the disease in DABEL, DASAC, DAGUA and DABEN, and regarding to the presence of comorbidities, the greatest significance was observed in DABEN, DAENT, DASAC and DAGUA. On the other hand, with regard to death in the population of elderly people affected by COVID-19 with SARS, a higher occurrence of this unfavorable outcome, comorbidities and the number of cases was observed in DABEL, DAICO, DAGUA and DASAC. And in these last four territories, considering the two populations compared, the highest numbers of NHIs and AMHDI were also found, as shown in Table 2.

**Table 2:** AMHDI and NHI associated with COVID-19 cases, comorbidities and deaths in elderly people with and without SARS by Administrative District, from 2021 to 2023.

ADs	AMHDI	NHI	Number of Cases		Comorbidities		Death	
			Without SARS	With SARS	Without SARS	With SARS	Without SARS	With SARS
**DABEL**	0.879	31	4,646	1,463	1,904	403	2,504	943
**DAICO**	0.645	3	2,211	163	1,128	80	902	100
**DAGUA**	0.693	5	4,766	184	1,954	78	2,030	91
**DASAC**	0.772	3	4,725	78	1,937	34	1,969	63
**DAENT**	0.644	1	3,857	47	1,967	30	1,484	39
**DABEN**	0.634	1	4,742	24	2,418	9	1,954	8
**DAMOS**	0.618	1	1,330	11	678	3	510	8
**DAOUT**	0.609	0	1,197	0	610	0	455	0

**Source**: EPIGEO/CCBS/UEPA

**Fig 1:**
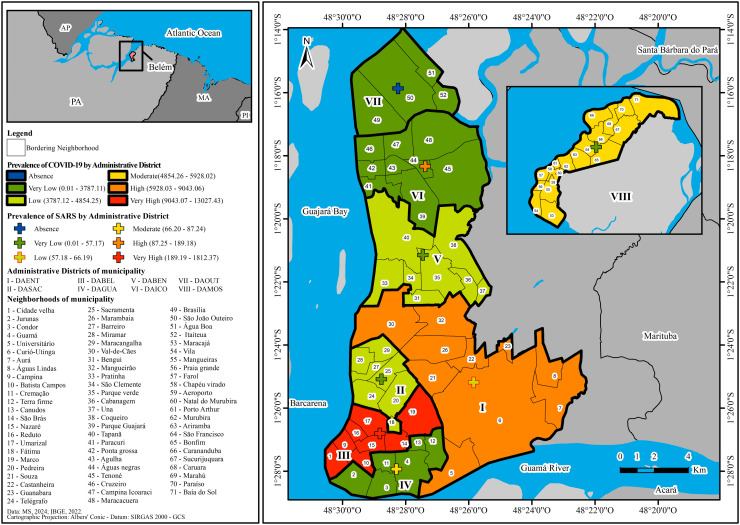
Spatial distribution of COVID-19 and SARS in the Administrative Districts of Belém, state of Pará, from 2021 to 2023. Source: EPIGEO/CCBS/UEPA.

The spatial analysis of the prevalence of COVID-19 and SARS in the ADs of the municipality of Belém considering the neighborhoods with the highest number of cases, showed that their distribution was non-homogeneous in these areas. Thus, with regard to the prevalence of COVID-19, it was observed to be: very high in DABEL (Marco); high in DAENT (Marambaia); moderate in DAMOS (Vila); low in DASAC (Pedreira) and DABEN (Coqueiro); and very low in DAICO (Campina de Icoaraci), DAGUA (Guamá) and DAOUT (São João do Outeiro). As for the prevalence of SARS, also in the ADs and neighborhoods with the highest number of cases, it was identified as: very high in DABEL (Marco); high in DAICO (Agulha); moderate in DAGUA (Guamá); low in DAENT (Marambaia); and very low in DAMOS (Vila), DABEN (Una) and DASAC (Sacramenta). However, the disease was not reported in any DAOUT neighborhood, as shown in Fig 1.

The spatial relationship between the occurrence of SARS, the AMHDI, the population quantitative and the presence of NHI showed that the prevalence of this disease is associated with the territorial variability of these variables in the study area and period. This means that in the ADs such as DABEL and DAICO, where very high and high prevalence was observed, the following stratifications were noted: very high and low AMHDI; low and moderate population quantitative; and very high and very low presence of NHI, respectively.

On the other hand, DAGUA and DAENT, which showed moderate and low prevalence had the following classifications: moderate and low AMHDI, and very high and low population quantitative, respectively. In these districts, there was a very low presence of NHI. Conversely, in DASAC, DABEN and DAMOS, where the prevalence of SARS was very low, the following classifications were identified: high, low and very low AMHDI, and high, high and very low population quantitative, respectively. In all of these territories, there was also a very low presence of NHI. Finally, in the DAOUT where no cases of SARS were notified, there was a very low AMHDI, a very low population quantitative and no NHI.

**Fig 2:**
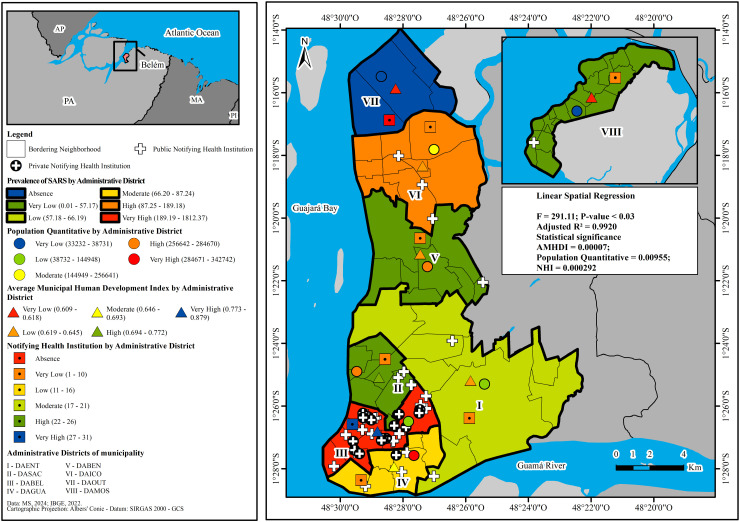
Spatial distribution of the prevalence of SARS and its relationship with population quantitative, Notifying Health Institutions, AMHDI in the Administrative Districts of Belém, state of Pará, during the period from 2021 to 2023. **Source**: EPIGEO/CCBS/UEPA.

The ordinary least squares multiple linear regression technique showed that the previously described spatial dependency relationship between the prevalence of SARS and its predictive factors (AMHDI, population quantitative and presence of NHI) was explained by 99.20%, with an adjusted R^2^ =  0.9920, using a statistically significant model of F =  291.11 and p-value <  0.03, as can be seen in Fig 2.

## Discussion

The fact that the highest occurrence of SARS was among males (53.23%) may be due to the historical and cultural practice of males not seeking preventive health services [20,21]. This situation is aggravated by the fact that they work long hours in a capitalist and patriarchal system, which has always placed greater responsibility for the economic viability of family life on men [22]. Furthermore, certain genetic characteristics make men more predisposed to developing the disease than women, such as having a greater amount of Angiotensin Converting Enzyme 2 (ACE-2) to which the coronavirus is linked [23]. These situations thus constitute risk factors for the greater occurrence of the disease in this gender.

The large number of cases of SARS in the elderly (56.11%) may be related to various situations, such as the advanced age of the patients associated with their immune status and ethnic characteristics, which may be correlated with some risk factors for this disease such as SAH and T2DM, especially in areas with precarious socioeconomic conditions and poor health service coverage [21,24]. In this age group, the progression of SARS-CoV-2 infection to the severe stage of the disease is related to the short incubation period of the virus, and there is a possible association with the absence of initial symptoms such as fever and leukocytosis, as well as the fragility of their respiratory system. These observations are found in other ecological and cross-sectional studies on the same subject in Brazil [25].

The observation that the majority of SARS cases declared themselves to be brown-skin (64.77%) might be tied to a cultural tradition influenced by their ethnic and evolutionary characteristics, which are particularly prevalent in the Amazon region. [16,24]. In the municipalities of the state of Pará, this population is part of a historical context of miscegenation of white, black and indigenous people, which occurred throughout the process of Portuguese occupation in the region [26]. Thus, according to the IBGE, around 80% of the population of the municipality of Belém calls itself brown, establishing a measurable causality when associated with various infectious diseases related to the environmental and socio-economic characteristics of where they live.

The higher percentage of persons with unknown schooling (71.77%) points to a weakness in the SARS notification service in terms of epidemiological surveillance, which may be due to the need for more efficient systematization of the process for obtaining, processing and analyzing data related to cases of the disease [27]. This is a risk factor for the management of health services, as it implies the need for accurate information aimed at effective decision-making processes focused on mitigating the disease. This situation has been observed in several Amazonian territories, especially in relation to infectious and parasitic diseases [14,28].

The observation that the highest percentage of cases occurred in urban areas (89.03%) is in line with the epidemiological profile of SARS described in other Brazilian cities. This scenario reflects major problems associated with Brazilian demography, which has been observed significantly with public health, due to the high concentration of people living in urban centers, especially in their outlying areas. This favors the occurrence of various infectious diseases, as has been observed in various studies carried out in the Amazon [29]. Other factors that may be conditioning this issue are those linked to differences in income, employment and the supply of health institutions and services, with a greater presence in urban areas to the detriment of rural areas. On the other hand, when considering a more localized scale, the relationship between the center and the periphery of Brazilian cities takes on particular significance, especially in the northern region of the country [15,30].

This situation historically marks the production and maintenance of structural and hierarchical inequality in Brazil, which is reproduced in municipalities such as Belém do Pará, due to its process of colonial occupation, one of whose main characteristics is the perverse logic of social, economic and demographic exclusion [15]. This situation has also been a conditioning for the rapid spread of the disease and its risk factors and is pertinent to a set of fundamental issues in society, such as the need for people to travel every day in search of work and public institutions with health services. This involves traveling from the outskirts to the urban areas where these are available, and usually includes using unhealthy means of transport in order to access these opportunities [29,31]. Thus, this situation engenders a relationship of privilege linked to the place where people live, which is maintained by the capitalist system’s interference in the social fabric, which has been observed on a recurring basis in Brazilian territories.

The high percentage of individuals with comorbidities (56.96%) may be related to the precarious state of their access to monitoring and control programs for SAH and T2DM, especially when these affect a fragment of the population characterized by men, the elderly, brown-skin people and those living in low-income areas [32]. Furthermore, when we consider that these diseases are risk factors for SARS, their occurrence can lead to an increase in the mortality rate, turning the individual into yet another number in an adverse statistic, which points to the need for mitigation. The association of these three pathologies thus constitutes a social and political injury that can be observed in the area of public health. In this context, it is essential to expand and intensify primary health care actions, with a view to reducing the occurrence of these chronic diseases as risk factors for worsening the clinical condition of COVID-19 as a syndemic, as observed in several Brazilian territories [33].

The higher number of “ignored” cases (62.15%) observed in the “vaccination” data item points to a problem related to monitoring and controlling the disease, especially with regard to epidemiological surveillance with efficient case records, since evaluations of immunization actions, as recommended in the National Immunization Program (NIP), may show evidence of contributing to reducing the occurrence of infectious diseases such as COVID-19, although the rate of vaccine abandonment is considered high nationwide [34,35]. In addition, the process of efficient notification of the disease can make it possible to carry out continuous analysis of the reinfection process, in order to avoid an increase in the number of SARS cases [36]. Information related to vaccination coverage, filled in correctly in the disease notification systems, has thus been important in reducing the severity of the disease, especially in populations with the risk factors discussed above, linked to age and the presence of comorbidities [36,37]. However, the implementation of immunization actions in the northern region of Brazil has been a major challenge for public health, especially considering the great distances, difficulty of access and logistical factors.

The fact that the highest percentage (62.69%) of SARS notifications were made by public NHIs reflects the importance such institutions have played in terms of attending to cases, to the detriment of private institutions that offered medium and highly complex services for treating this pathology, such as mechanical ventilation, administration of specific drugs used in intensive care, physiotherapeutic devices and multi-professional actions [38]. This situation has taken on historical importance in the country, especially when we consider the recent adverse political scenario, observed in narratives such as that of the minimal state, which advocated the dismantling and weakening of public health services [39,40]. This is especially relevant in the context related to the COVID-19 pandemic, where the high demand for health care resulting from its evolution into the severe form of the disease, showed the need to reverse this scenario, and engendered negative discourses that involved highly biased and arbitrary disinformation [41].

From another perspective, the political decision to increase the supply of public services for SARS care was a metaphor for the republican and democratic guarantee of access to care for this disease, as a right for all Brazilian citizens in a universal, comprehensive and equitable way, regardless of their socioeconomic status, especially during the critical period of the pandemic. Thus, the majority of notifications were made by public SARS care institutions, mainly for the low-income population, which is the majority of the population. However, it is important to note that access to these services was insufficient and sectorized, despite the magnitude of the number of cases observed in the municipality of Belém. This situation was emblematic of the need to expand and intensify the actions carried out by SUS in the study area, reflecting its demand to strengthen its health services considering the regional demographic and socio-economic specificities, especially those related to unequal access for the low-income population, which was the hardest hit by the disease, in line with the national trend [42,43].

The quantitative significance of the SARS cases that ended in death (49.87%) may be associated with various factors, such as political, demographic and socioeconomic issues [44,45]. From the point of view of the political decision to provide equipment to deal with the disease, the precariousness of the peripheral area of the municipality was identified in relation to the coverage of basic, secondary and tertiary health care in it, with low hiring and distribution of specialized human resources, in addition to the lack of reorganization of the municipal health network, following the adverse scenario observed in other Brazilian territories [40,42]. This has resulted in the emergence of large areas of gaps in care and a significant difference between the supply of public and private beds in Intensive Care Units (ICUs) in all ADs except the central one.

The fact that the highest occurrence of death in the elderly population with COVID-19 with and without SARS was observed mainly in DABEL, DASAC and DAGUA may be due to their notification characteristics and their relationship with the number of cases, AMHDI, NHIs and associated comorbidities. This scenario may be due to the relative combination of higher average per capita income and jobs in these three territories, as well as the greater supply of health services for the disease such as diagnosis, treatment and medication, the prevalence of SAH and T2DM, where DABEL is considered central and the others adjacent. This situation follows the profile of the disease observed worldwide and others infectious diseases caused by viruses [12,19,24,46,47].

The scenarios described above are diametrically opposed to the ones observed in districts considered peripheral, such as DAMOS and DAOUT, in which the low notification of cases of death in the elderly, especially with SARS in these two districts, was also aggravated by the geographical characteristics of their insular areas such as Cotijuba, Mosqueiro and Caratateua, whose native population is considered rural. These people have historically been disadvantaged by the lack of infrastructure for accessing health services due to the great distance and complex path from their homes to them, especially as they are located on the mainland of the municipality [12]. These populations have a primary production process based on small-scale fishing, extractivism and subsistence agriculture associated with low income and precarious education, as well as dispersed housing in a natural environment with low levels of human impact. These risk situations are described in various works on demography, health and socioeconomics, such as the 2020 municipal yearbook and other sources [48].

The informational absenteeism related to the low occurrence of deaths in the elderly population with SARS living in these districts made up of island and mainland areas was conditioned by the socioeconomic, geographical and public policy characteristics of their places of residence. This situation was aggravated by the individual characteristics linked to the lifespan of these elderly people with their physiological and psychosocial frailties. In this context, we highlight the establishment of territorial and social inequalities that are also reflected in the disproportionate presence of public and private healthcare facilities such as hospitals, pharmacies and urgent and emergency care units aimed at tackling COVID-19 in the population of elderly people with and without SARS, observed in rural and urban areas of the municipality [33,36].

With regard to the demographic factor, the situation in the municipality of Belém, with approximately 12% of its population living in its central area, where most of the services aimed at dealing with the disease are concentrated revealed a systemic inequity in health related to the low supply of services in the areas where they are most in demand, i.e., in the peripheral ADs [48,49]. This situation resulted in suffering of around 88% of the Belém population, who live in densely populated areas, due to the difficulties for the search for assistance for COVID-19, considering the risk for SARS. Thus, this adverse scenario associated with the precarious supply of services in a regionalized manner and its relationship with the municipal demographics may have influenced the unfavorable outcome of patients affected by the disease, with a high percentage of deaths in the municipality [48,50]. This complex scenario shows the multifactorial dimension of the pandemic and its adverse consequences.

The socio-economic differences between the ADs of Belém do Pará associated with the outcome of death from SARS in these districts have assumed significant importance in the adverse epidemiological scenario of the disease, considering the high concentration of wealth in this municipality [51–53]. This can be seen notably in the relationship between the district located in the central zone (DABEL) and the others in the periphery, where the income gradient can be up to 20 times lower [54,55]. This situation has become worrying over the course of the pandemic, especially given the social inequalities that exist in neighborhoods on the periphery, such as the presence of more than 50% of their territory having sub-standard housing or slums, as well as population densification, low provision of public health services, large numbers of people in a process of social vulnerability and other risk factors for the occurrence of SARS [50,52,53].

Observing the non-homogeneous spatial distribution of the disease correlated with the variability of population quantitative, the AMHDI and the supply of NHIs in the ADs studied reveals its uneven development history, especially when we consider that this region was colonized approximately 400 years ago. Over this period, the municipality has expanded in a disorderly fashion, with migratory flows that have intensified over the last 50 years [56,57]. In this context, the spatial differences observed between the demographic, socioeconomic and public health policy variables categories that were analyzed reflect the scenario of social inequities to which residents of vulnerable areas are subjected. This situation shows a systematic process of socio-spatial segregation of the population in the face of the risks of the disease, which was marked by the dependence of the peripheral area on the central area of the municipality [50,53,58].

These relationships observed in a territorialized context were caused by the lack of public services and employment opportunities in the peripheral ADs and more markedly in the central districts of the municipality, both from a quantitative and qualitative point of view, establishing a socio-spatial process of producing risk factors for SARS [50]. The epidemiological silence marked by the absence of cases in the DAOUT may be associated with the high percentage of incomplete information related to the patients’ place of residence, despite the obligation of properly filling out the notification form recommended by ministerial ordinance 1,792 of July 17, 2020 [40]. Thus, surveillance of the disease in the study area still falls short of providing an efficient monitoring and controlling service, principally considering Health Information Systems usage. This is in line with the national scenario related to the problem of underreporting of data in these digital environments, notably in light of the precariousness of the existing technological infrastructure in the Amazon [59,60]. Given the above, it was possible to highlight the spatial relationship between the variables analyzed and the worsened outcomes in the evolution of COVID-19 to the severe form of SARS in the peripheral areas of the municipality.

It should be noted that this study involved several limitations related to performing more contextualized analyses of the variables studied, given the significant amount of unknown data and the lack of information on people who fell ill with COVID-19 and had SARS, especially in areas experiencing socioeconomic vulnerability and public health policy inadequacies during the pandemic. This situation points to the need to strengthen epidemiological surveillance of the disease in the municipality, in order to report it more efficiently. This situation has been observed in several other studies on this subject in the Amazon [61,62].

## Conclusions

In general, the epidemiological profile of SARS in Belém do Pará followed the pattern observed nationwide and was statistically significant. The analysis of the occurrence of the disease revealed a spatial and socioeconomic segregation of the population in the ADs studied. This situation was observed mainly in the low supply of health services aimed at treating the disease especially for the elderly population, in addition to the precarious living conditions and the large number of residents in the peripheral neighborhoods to the detriment of the central ones of the municipality. This scenario characterizes a process of unequal territorial development, marked by the logic of dependence on public health policies, as well as the need to address the liabilities of work, education and income, especially in the places with the vulnerabilities identified. It is therefore important to emphasize the urgency of expanding the services described above, through affirmative action to ensure equal access to them in the study area.
